# A systematic review of AI-driven intelligent tutoring systems (ITS) in K-12 education

**DOI:** 10.1038/s41539-025-00320-7

**Published:** 2025-05-14

**Authors:** Angélique Létourneau, Marion Deslandes Martineau, Patrick Charland, John Alexander Karran, Jared Boasen, Pierre Majorique Léger

**Affiliations:** 1 Department of Teaching at Université du Québec à Montréal (UQAM), Montréal, QC Canada; 2 Department of Information Technologies at école des Hautes Études Commerciales (HEC), Montréal, QC Canada

**Keywords:** Education, Education

## Abstract

The use of artificial intelligence in education (AIEd) has grown exponentially in the last decade, particularly intelligent tutoring systems (ITSs). Despite the increased use of ITSs and their promise to improve learning, their real educational value remains unclear. This systematic review aims to identify the effects of ITSs on K-12 students’ learning and performance and which experimental designs are currently used to evaluate them. The 28 studies analyzed in this systematic review included a total of 4597 students (*N* = 4597) and used quasi-experimental designs with varying intervention durations. Overall, our findings suggest that the effects of ITSs on learning and performance in K-12 education are generally positive but are found to be mitigated when compared to non-intelligent tutoring systems. However, additional research with longer interventions and increased sample sizes with greater diversity is warranted. Additionally, the ethical implications of using AI for teaching should be investigated.

## Introduction

According to the United Nations Educational, Scientific and Cultural Organization, there is a worldwide educational crisis, with more than 250 million children out of school and an additional 600 million failing to reach minimum competency levels^1^. UNESCO’s Sustainable Development Goal 4 states that sustainable development can only be achieved by leveraging the potential of the digital revolution, which increasingly encompasses artificial intelligence (AI)^2,3^. Research initiatives are encouraged to investigate how new technologies can impact teaching and learning and how they can be used ethically and responsibly in education.

Existing research emphasizes the significance of providing learners with 21st-century skills, including the more effective use of digital technologies for learning^3–5^. Educational resources and learning activities are increasingly disseminated to students through digital learning environments^6^. These environments are designed to provide education adapted to the user’s characteristics, needs, and behaviors. The use of AI in educational resources is a growing industry that promises to transform education by creating tutoring systems that could personalize learning.

In this regard, the use of AI in education (AIEd) has experienced substantial growth over the past decade. AIEd encompasses a broad range of applications, from adaptive learning platforms that curate customized content to fit individual learning styles and paces^7^, to AI-driven analytics tools that forecast student performance and provide educators with actionable insights^8^. Developments in AIEd have expanded the educational toolkit to include chatbots for student support, natural language processing for language learning, and machine learning for automating administrative tasks, allowing educators to focus more intently on teaching and mentoring^9^. Due to technological convergence, these tools have evolved into multipurpose, generative pre-trained transformers (GPTs). GPTs, such as OpenAI’s GPT-4, Anthropic’s Claude, and Google’s Gemini, are large language models (LLMs) that combine extensive language datasets with immense computing power to create intelligent models that, after training, can generate complex, advanced, human-level output^10^ in the form of text, images, voice, and video. These models are capable of dynamic human-computer dialogs, continuously responding with novel output each time users input a new prompt, having been trained on data from the vast corpus of human knowledge, spanning the physical and natural sciences to medicine and pedagogy. Therefore, It is likely that AIEd will continue to be a crucial topic in the coming years^11,12^.

Computer tutoring was first introduced in classrooms in the late 1960s and has since undergone significant improvement, often incorporating advancements in AI. Those updated tutoring systems are now generally referred to as Intelligent Tutoring Systems (ITSs)^13^. ITSs are software programs generally equipped with AI programming that can detect, comprehend, and adapt to the learner’s progress. They monitor student progress, identify difficulties and errors, navigate structured subject content to offer and tailor the difficulty level, thus developing an optimal path for learning^3^. One well-known example of an ITS is Duolingo^14^, a mobile application for language learning that personalizes instruction for each user^14^. ITSs are becoming more prevalent in modern society, supporting traditional educational environments like classrooms and alternative learning contexts such as distance learning or professional training. According to UNESCO’s 2021 Guidance for Policy-makers, they are currently the most extensively studied application of AIEd^15^.

However, while there is limited literature highlighting the effects of ITSs on K-12 education, studies on ITSs often conflate the effects on children’s and adults’ learning or on learning in school versus professional settings. Despite the growing use of ITSs and the potential they offer to the education industry, stakeholders still struggle to navigate between the potential to improve learning outcomes and reduce inequities in education and the actual educational value, which remains uncertain^16^.

In 2016, Kulik and Fletcher conducted a meta-analysis of 50 controlled assessments of ITSs^17^; their findings revealed a lack of scientific consensus regarding their effectiveness. However, they also demonstrated that ITSs can be highly effective instructional tools. While many studies have suggested that the effect of ITSs on learning could surpass that of a human tutor^13^, others have raised questions^18^, and highlighted the limitations of ITSs^15^. Another systematic review of AI applications in higher education emphasized the necessity for research on the effectiveness of ITSs^12^. This review noted that the positive outcomes of using new technologies in the classroom are almost never attributed to the novelty effect^12^, even though it has been shown that novelty in itself can improve students’ memory and learning^19^.

With this said, a recent analysis by Honebein and Reigeluth^20^ speaks to how ITSs can be highly effective, but only when they embody sound pedagogical features applied under the right conditions. They identified that key features such as immediate feedback, guided practice, and adaptivity are not simply superfluous additions – they are grounded in decades of instructional theory and have demonstrable positive effects on learning. Moreover, the effectiveness of these features can be maximized in certain situations, such as domains and contexts that truly leverage individualized, active learning. They state that comparing a well-established “traditional” learning method to a half-formed ITS (lacking these features) is both unfair and uninformative. Their central thesis is that to “improve” rather than “prove” ITS effectiveness, designers of ITS must implement proven features and clarify the situational variables for their use; they claim that when an ITS is well-aligned with instructional theory – applying the correct methods for the right learners under the right conditions – it consistently produces positive outcomes.

In this regard, one study that investigated the effects of an ITS longitudinally was performed by Pane and colleagues^21^. In this large-scale randomized controlled trial, they evaluated the Cognitive Tutor Algebra I (CTAI), which provided individualized instruction to address students’ specific needs. The CTAI utilizes a multi-modal approach, including diagrams, equations, text, and concepts that were contextualized in real-world problem scenarios. They demonstrated the effectiveness of CTAI in improving student algebra proficiency. This improvement only emerged after sustained implementation, such that they observed notable improvements in the second year of the study, particularly in high schools, where students outperformed control groups with an effect size of approximately +0.20 standard deviations. This effect, they claim, is comparable to the benefit of an additional year of algebra instruction. However, the study also showed that the use of the CTAI in middle schools exhibited a similar but non-significant trend in learning outcomes. Overall, the results of this study suggest a certain consonance with the thesis of Honebein and Reigeluth^20^, in that, the correct features were deployed in the ITS, but the situational variables for use were only met in high schools and not middle schools. A case of the right tools for the right learners, of course, other confounding factors may have affected the results, such as issues arising within the initial implementation period during which teachers and schools adapted to the ITS and the blended learning model, which may have differed between school tiers.

Smith and Sherwood noted that researchers have been striving to develop computer tutors as effective as human tutors since computers were first developed^22^. VanLehn^13^ and Kulik and Fletcher^17^ have contributed to the understanding of the general effectiveness of ITSs compared to other learning methods, such as human tutoring or no tutoring, regardless of the learning environment (adult training, school environment, etc.). In contrast to the results of Pane et al.^21^, Kulik and Fletcher^17^, in their review of ITS, indicated that only three studies were conducted in school settings and found no real improvement in school (K-12) performance due to the deployment of ITS. This finding emphasizes the importance of conducting a systematic review encompassing both the effectiveness and improvement of ITS in primary and secondary (K-12) education, similar to Zawacki-Richter et al.’s review of AI applications in higher education^12^. The aim is to provide guidance to stakeholders at all levels concerning the development, deployment and use of ITS in education.

Thus, the gap in scientific knowledge related to ITSs goes beyond their efficacy in providing positive learning outcomes to include what features and situational variables are beneficial to the successful deployment of ITS. As such, comprehensive research is needed to address the application and effects of AIEd in primary and secondary (K-12) education. Despite numerous years of research and case studies on the implementation of ITS, little is known about their effect on the quality of learning^16^. Additionally, there is currently no systematic or generalizable understanding of how to apply other forms of AI to optimize learning outcomes^3^.

While the ethical implications of AIEd in the broader sense are beyond the scope of this review, we understand that there is a rich and developing literature in this area. Broadly speaking, from an ethics standpoint, stakeholders should ensure that ITS systems deployed in educational environments are fair, equitable, transparent and beneficial to learners^23^. Ethics in AIEd are linked to multiple ethical dimensions such as fairness, responsibility, transparency, accountability, agency, interpretability and explainability^24,25^, which can potentially make an AI application more trustworthy and accepted.

This systematic review aims to evaluate current and recent advances in ITS research enabled by AI innovation and ultimately address the following questions:

1. What experimental designs are used to evaluate the effects of ITSs?
2. What are the effects of ITSs on K-12 students’ learning and performance?

## Results

Table 1 outlines the included articles, study location, a sample description, the intervention duration and the controlled variable. As previously stated, two of the articles each contained two studies. To differentiate between the studies within an article, each one was labeled as [a] and [b], for example: Cui et al. [a].

**Table 1:** Included studies

Authors	Country	Sample size	School level (grade)	School subject	Intervention Duration (weeks)	Controlled variable
Chen and Huang^29^	Taiwan	160	8	Computer science	7	ITS/Teacher
Long and Aleven^44^	United States	122	8	Math	1	ITS/modified ITS
Özyurt et al.^45^	Turkey	25	10	Math	8	No control
Long and Aleven(a)^46^	United States	98	8	Math	1	ITS/modified ITS
Long and Aleven(b)^46^	United States	56	7	Math	0	ITS/modified ITS
Roscoe et al.^27^	United States	113	10	First language	25	No control
Dolenc et al.^30^	Slovenia	58	8	Science	1	ITS/Teacher
Choi^31^	Korea	124	High school and middle school	Second language	4	ITS/Teacher
Jordan et al.^32^	United States	72	11	Physics	1	ITS/(non-intelligent) TS
McCarthy et al.^33^	United States	234	High school	Science	1	ITS/modified ITS
Bernacki and Walkington^34^	United States	150	9	Math	16	ITS/modified ITS
Holstein et al.^49^	United States	286	7; 8	Math	20	ITS/modified ITS
Cui et al.(a)^28^	China	163	8	Math	1	ITS/Teacher
Cui et al.(b)^28^	China	104	8	Second language	1	ITS/modified ITS
Walkington and Bernacki^35^	United States	106	9	Math	0	ITS/modified ITS
Chen et al.^47^	Taiwan	24	4	First language	1	No control
Huang et al.^50^	United States	129	9	Math	4	ITS/modified ITS
Katz et al.^36^	United States	73	High school	Physics	1	ITS/(non-intelligent) TS
Ingkavara et al.^37^	Thailand	292	High school	Physics	4	ITS/(non-intelligent) TS
Ökördi et al.^53^	Hungary	810	3; 4	Math	5	ITS/Teacher
Vest et al.^51^	USA	167	6; 7; 8	Math	1	ITS/modified ITS
Wijekumar et al.^38^	USA	464	5	First language	6	ITS/Teacher
Borchers et al.^39^	USA	82	9	Math	1	ITS/Teacher
Nehring et al.^40^	USA	100	12	Math	30	ITS/Teacher
Tang et al.^41^	China	65	10	Math	1	ITS/(non-intelligent) TS
Horvers et al.^42^	Netherlands	114	5	Math	1	ITS/modified ITS
Uriarte-Portillo^48^	Mexico and Spain	106	9	Math	2	ITS/(non-intelligent) TS
Khasawneh^43^	Saudi Arabia	300	High school	Math	8	No control

*n.a.* not available.

It is worth noting that the table does not include effect sizes. Indeed, although we recalculated these Cohen’s d values using the available information (means, standard deviations, group sizes, eta-squared, etc.), we observed that the research designs vary so much from one study to another that it becomes complex and not very relevant, for comparison purposes, to present the information in a clear and concise manner.

Also, It is important to mention that an additional article by Roscoe and McNamara^26^ described the same study as another article by Roscoe et al.^27^ albeit with less detail. For the sake of this review, both articles were considered as one study.

### General overview

Ninety-six percent of the articles were authored by individuals affiliated with educational science, computer science, or both. Only one article was authored by individuals affiliated with the studied ITSs’ company^28^. Of all the articles included, 62% were authored by individuals with an educational science background^29–43^. Fifteen percent were authored by individuals with a computer science background^44–48^. The remaining articles were authored by individuals from both backgrounds (19%)^27,28,49–51^.

It is encouraging that the majority of authors are from the fields of education or computer science, with fewer from ITS companies. This enhances the reliability of the results regarding student learning and performance in these studies.

This result differs from the systematic review of ITSs research in higher education conducted by Zawacki-Richter et al.^12^ In their review, only 8.9% of the included articles were written by authors with an educational science background, while the others were written by authors with Computer Science and STEM backgrounds. This discrepancy could arise from the use of different databases, namely EBSCO Education Source, Web of Science, and Scopus, or from the fact that Zawacki-Richter’s review covered all AI applications, whereas the current specifically targeted ITSs. ITSs are primarily employed in traditional and alternative educational settings within the field of AIEd.

The publication rate of the included articles shows signs of slight increase in recent years. Given that ITSs are regarded as the most prevalent and highly sought-after educational application of AI, attracting considerable investment and interest from technology companies^15^, we anticipated a higher number of studies meeting our criteria. For comparison, Zawacki-Richter et al. reported 29 studies investigating ITSs in higher education from 2007 to 2018^12^. Similarly, this variation could stem from the difference in databases used. However, it may also result from the current review solely concentrating on the impacts of ITSs on learning and performance rather than other educational variables such as interest, attitude or motivation towards learning. Additionally, it could be due to the feasibility of conducting studies in higher education compared to K-12 settings involving minors. Nevertheless, the effects of ITSs on learning and performance have continued to be a subject of interest over the past decade. There has been a consistent publication trend, with one or two articles published annually from 2011 to 2016, followed by an increase to two or three articles each year since 2017.

This review included contributions from eight countries, with a notable concentration in the USA and Asia. Most articles originated from the USA (54%)^27,32–35,38–40,44,46,49–51^. The remaining articles primarily originated from Asia (27%), including Taiwan^29,52^, China^28,41^, Thailand^37^, Korea^31^, Turkey^45^, and Saudi Arabia^43^, while only four (15%) came from Europe (Slovenia, Hungary, Spain and Netherlands)^30,42,48,53^.

None of the articles included in this review mentioned any consideration of AI ethics. This lack of attention on ethical concerns in studies investigating the effects of ITSs on student learning and performance prompts questions regarding the extent to which educators and researchers have addressed the ethical implications associated with the use of AI in education. This oversight highlights the need to thoroughly examine the ethical implications of the widespread use of intelligent tutoring.

### What experimental designs are used to evaluate the effects of ITSs?

The effects of ITSs have been studied using various experimental designs across diverse educational contexts, including different school levels and subjects.

Educational context: The school level and the subject were compared to assess differences in educational context. Roughly half of the studies (54%)^27,32–37,39–41,43,45,48,50^ involved high school students and one included both high school and middle school students^31^. Nearly all the remaining (32%) involved middle school students^28–30,44,46,49,51^. Only four studies (14%) involved elementary school students, and none were conducted with preschool groups^38,42,47,53^.

Most of the studies (82%) were carried out in subjects related to STEM^28–30,32–37,39–46,48–51,53^, while the others were focused in Language arts, first^27,38,47^ or second^28,31^ language. This finding was consistent with the research conducted by Holmes and Tuomi^16^, and UNESCO,^15^ which suggested that ITSs are well-suited for subjects with a structured approach, such as mathematics or physics. This may explain why studies have primarily focused on higher education, where these subjects are taught.

**Fig. 1:**
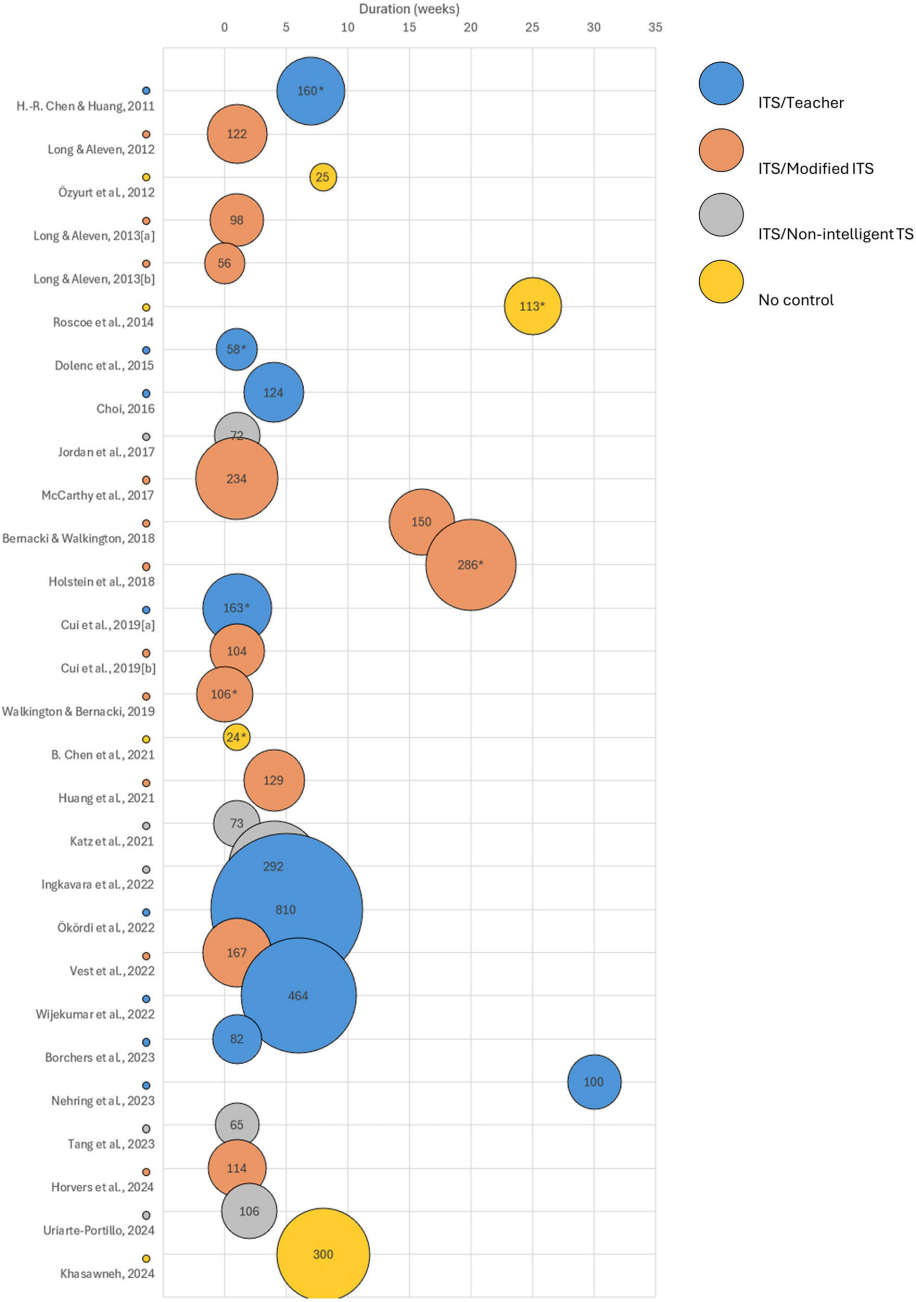
Comparison of studies based on sample size, type of control group, and duration of the study. Bubble size indicates the sample size. An asterisk indicates studies that included the effect sizes of their results.

Experimental designs: Fig. 1 shows that researchers mainly utilized quasi-experimental methods in most cases^27–38,44–47,49,50^. These methods involved an experimental group using an ITS, while a control group used an alternative intervention to learn the same subject. Effects were measured with a pre- and post-test administered to both groups. In their meta-analysis of ITSs, Kulik and Fletcher observed that the effect measure depended on the nature of the tests, whether they were locally developed or standardized^17^. The difference between locally developed and standardized tests is worth noting, as it affects the interpretation of educational outcomes. This suggests that the context and design of the assessment tools may influence the measurement of educational effects. This also suggests that alignment of the test and the instructional aims are critical determinants of assessment results. As not all studies included in this review have the same type of control group, the studies were categorized into four groups based on their control group type (as listed below and in Fig. 1). This was done to analyze the effects of ITSs on students’ performance. The four types of control group were:

- ITS vs Teacher (8 studies)^28–31,38–40,53^: The control group received a traditional, non-digital teaching on the same concepts as the experimental group.
- ITS vs Non-intelligent tutoring system (TS) (5 studies)^32,36,37,42,51^: A digital learning environment without artificial intelligence was used in the control group. It is noteworthy that all these studies took place in high school physics classes.
- ITS vs Modified ITS (11 studies)^28,33–35,41,44,46,48–50^: The control group used a modified or older version of the ITS tested by the experimental group.
- ITS vs No control (4 studies)^27,43,45,47^: There was no control group. This category included a qualitative study^45^, an implementation study^27^, and a study on gender differences in performance^47^.

Intervention Duration: Fig. 1 illustrates that half of the interventions lasted less than a week^28,30,32,33,36,39,41,42,44,46,47,51^, with some as brief as a single class period^35,46,51^. The International Brain Research Organization (IBRO), in partnership with UNESCO’s International Bureau of Education (IBE), suggested that novelty can improve students’ memory and learning^19^. Considering this, it remains unclear how one can draw conclusions about long-term effects on students’ performance from such brief interventions. Can these effects be attributed to the ITS itself or simply to the novelty aspect of it? Some other studies lasted several weeks^27,29,31,34,37,38,40,43,45,49,50,53^, limiting the novelty aspect of the intervention. The longest intervention lasted 30 weeks and was conducted with a control group^39^.

### What are the effects of ITSs on K-12 students’ learning and performance?

As previously mentioned, studies were categorized into four groups based on experimental design. However, the included studies do not provide many effect sizes.

#### ITS vs teacher

Seven out of eight studies comparing an ITS to traditional or usual teaching reported a significant positive effect of ITSs on student performance, with effect sizes ranging from medium to large. One study^39^ found no significant difference between traditional teaching graphs and ITS use.

Thus, Cui et al. [a] compared the Yixue Squirrel AI ITS to traditional offline teaching methods regarding the Pythagorean theorem over a three-day period, comprising a total of five hours of learning^28^. A total of 90 students used the ITS in the experimental condition, while 73 were in the control condition. According to Cui et al., the learning gains were 4.19 times greater for the experimental group compared to the control group, with a medium-sized effect (Experimental group *M* = 9.38, *SD* = 11.08; Control group *M* = 1.81, *SD* = 10.91; Hedges’s *g* = 0.68; *F*(1.160) = 16.80, *p* < 0.001, partial η^2^ = 0.10)^28^.

In the study by Chen and Huang, 160 computer science students were taught how to use the Internet, Word, and PowerPoint^29^. In this experimental condition, 81 participants used an unnamed ITS designed specially for this study, while 79 were assigned to the control condition, receiving traditional teaching methods. The ITS was used for seven weeks, but there is no further indication of the actual time devoted to learning with or without the ITS. Learning gains were measured using pre- and post-tests. A significant difference in the experimental and control groups’ test results was shown by one-way analysis of variance (ANCOVA) (Experimental group *M* = 68.889; Control group *M* = 64.621; *F* = 4.272; *p* < 0.05)^29^.

In a study conducted by Choi, 32 high school students and 30 middle school students used ITS iTutor, a tool designed to teach English as a foreign language, for eight ninety-minute sessions over four weeks^31^. The control group comprised an equal number of students taught the same grammatical concepts in a traditional, teacher-centered, paper-based setting^31^. The study found a statistically significant difference between the experimental and control groups in the pre- and post-tests, demonstrating the effectiveness of the ITS as shown by a two-way ANOVA (*F* = 234.344 and *p* < 0.05)^31^. The ITS had varying effects on middle and high school students as a statistically significant interaction effect between the experimental groups and the education level was reported (*p* = 0.013; α = 0.05). This finding suggested that students react differently to the tutoring program depending on their education level, middle school or high school, and between the control and experimental groups^31^. In particular, middle school students showed greater improvement in the ITS condition and benefited more from it compared to high school students. Notably, this type of comparison between levels of education is rare in the literature. This study suggested that educators may need to differentiate ITSs depending on the level of education to effectively increase student’s performance.

Wijekumar et al. conducted an experiment with 5th-grade students from 7 different schools using the We Write ITS over a 6-week period^38^. The study consisted of two parts, but only the first part, which used an ITS, was considered here. The study aimed to investigate planning skills and writing quality, which were assessed using one pre-test and two post-tests. The experimental group (*n* = 299) consisted of 194 students who took the writing quality pre-test, 193 who took the planning pre-test, and 145 who completed both post-tests. The control group (*n* = 165) consisted of 127 students who took the writing quality pretest, 126 who took the planning pretest, and only 9 who completed both post-tests. To understand these numbers, it is important to acknowledge that some students took only the pre-test, not the post-test, and vice-versa. Although the small control sample may have been due to the teacher’s reluctance to allocate instructional time for further assessments, as stated by the authors, it limited the ability to have a normal statistical sample^38^. The authors reported a significant medium-sized effect size (*d* = 0.77) on the planning skills of the students in the ITS compared to those in the control^38^. The authors mentioned that classes with lower initial writing quality scores seemed to benefit from the ITS more than classes with higher initial scores, although the effect on writing quality was small and not statistically significant. It is noteworthy that the authors of this study emphasize that the ITS enhances but cannot replace teacher-led instruction and that teachers should receive adequate training regarding the use of computer tools^38^.

Dolenc et al. did not directly compare the performance of students who used an ITS with a control group, but rather with national standardized test results^30^. Fifty-eight students used the TECH8 ITS for two 45-minute sessions to study the gear subject and then underwent a summative assessment of knowledge comparable to the National Assessment of Knowledge (NAK) in Technology and Science for the years 2008 and 2010. Their results were compared to the national results of the 2008 and 2010 NAK, revealing a large effect size of the ITS (2008: *d* = 0.99; 2010 : *d* = 1.30). The authors suggested that the TECH8 ITS attained better outcomes compared to traditional teaching methods^30^. The ITS results were also comparable to those of other ITSs, although the authors did not specify any particular ITSs.

Ökördi et al.^53^ conducted a quasi-experimental study on 2187 students from third and fourth grade. After excluding students with more than 50% missing data on a test or those who did not meet the minimum participation criteria, the final sample comprised 810 pupils who completed a pre-test, a post-test, and a follow-up test three months later on multiplication and division. This included 414 students in Grade 3 and 396 in Grade 4, equally divided between intervention and control groups in a manner that mitigated school-related factors. After the pre-test, both conditions groups had classroom lessons, and the intervention group combined those lessons with sessions on the eDia online platform. The intervention lasted four to six weeks and took place in the school during regular school hours and each online session took approximately 10–20 min. According to Ökördi et al., students who completed more than half of the online sessions improved their skills by one-third of a standard deviation, while the control group’s progress was only half that amount^53^.

Nehring et al.^40^ conducted a study on the ALEKS PPL web-based mathematics learning platform in 12th grade across five different schools, with a total of one hundred students. Students from two schools constituted the control group, receiving only traditional classroom lessons (*n* = 27). Students from the other three schools formed the intervention group (*n* = 73), combining traditional classroom lessons with modules on the ALEKS platform. Both groups completed the ALEKS PPL Mathematics Placement Exam in October and again in May, and the data was combined with data from the online platform in a 2 × 2 mixed ANOVA (F(1, 98) = 19.16, η² = 0.16, *p* < 0.001). The results indicated that students in the intervention group significantly increased their exam scores between October and May (*M*_diff = 13.55, *SE* = 1.72, *p* < 0.001, *d* = 0.87, 95% *CI* [10.14, 16.96]), whereas the control group exhibited no statistically significant change in mean performance (*M*_diff = −0.93, *SE* = 2.83, *p* = 0.744).

In Borchers et al.’s^39^ study, 82 9th-grade mathematics students used the ITS Mathtutor to learn three units on linear graphs. The students completed a pre-test on all units on Day 1, learned two units on Day 2, completed a first post-test on those two units on Day 3, learned the last unit on Day 4, and completed a second post-test on the last unit on Day 5. Each test was completed in two different formats (paper and ITS); half of the groups answered the paper test first (PT), while the other half did the tutor test first (TP). The students were divided into four conditions, alternating between paper and tutor learning and testing. Paired t-tests showed statistically significant learning gains for both the paper and ITS conditions (tutor: *t*(281) = 2.76, *p* < 0.001; paper: *t*(287) = 7.94, *p* < 0.001). An ANOVA showed that learning gains were similar in both the ITS (*M* = 0.13, *SD* = 0.30) and paper conditions (*M* = 0.15, *SD* = 0.31) (F(2, 222) = 5.28, *p* = 0.006). There was a significant interaction between the condition and the learning unit, favoring paper for one unit and ITS for another unit. There was also a significant main effect, indicating that learning gains were twice as high when the test format matched the practice environment (*M* = 0.18, *SD* = 0.30) compared to when there was no such match and students had to transfer knowledge across formats (*M* = 0.10, *SD* = 0.30) (*t*(567.96) = − 3.28, *p* = 0.001).

#### ITS vs non-intelligent TS

Four studies compared an ITS to a non-adaptive or non-intelligent tutoring system in high school physics classes^32,36,37^. One study reported positive learning gains with the ITS^37^, while the other three reported no significant difference in learning gains between the ITS and the non-intelligent tutoring system^32,36,41,48^.

Ingkavara et al. conducted a study with an experimental group of 144 students who participated in a self-regulated online learning approach guided by personalized learning, supported by an unnamed ITS^37^. The control group of 148 students followed a conventional self-regulated online learning approach without guidance from a teacher. Both groups studied electric circuits for a month^37^. Learning gains were significantly higher in the experimental group (*M* = 7.37, *SD* = 2.237) compared to the control group (*M* = 6.07, *SD* = 1.908): (*t*(290) = 5.350, *p* < 0.05)^37^. This is the only study in this category that reported significant results.

Jordan et al. conducted a study with 37 students in the experimental group and 35 in the control group^32^. Both groups used the Rimac system for one class period, specifically on the kinematic subject^32^. In the control version, the tutoring system broke down each step, regardless of the student’s prior knowledge while, in the experimental version, the ITS only broke down the necessary steps into sub-steps based on the student’s prior knowledge of the content^32^. The authors reported no significant effect difference between both groups, suggesting that students learned the same regardless of their assigned group^32^.

Katz et al.^33^ reported two studies: Jordan et al.’s^32^ study, presented above, and Albacete et al.’s.^53^ In the study by Albacete et al., the Rimac system was used over a four-day period.^54^ The 31 students in the experimental group used an adaptive version of the system, while the 42 students in the control group used a non-adaptive version.^54^ The study found no significant difference in learning gains between the two conditions when controlling for students’ prior knowledge (F(1.70) = 1.770; *p* = 0.19).^54^ An additional independent samples t-test showed no significant difference between mean learning gains in the experimental and control groups (Experimental group M = 0.087, SD = 0.074; Control group M = 0.112, SD = 0.096; t(71) = 1.226, *p* = 0.22). However, an analysis of variance (ANOVA) revealed that students in the experimental group learned significantly faster, irrespective of their prior knowledge of the content. reported two studies: Jordan et al.’s^32^ study, presented above, and Albacete et al.’s^53^. In the study by Albacete et al., the Rimac system was used over a four-day period^54^. The 31 students in the experimental group used an adaptive version of the system, while the 42 students in the control group used a non-adaptive version^54^. The study found no significant difference in learning gains between the two conditions when controlling for students’ prior knowledge (*F*(1.70) = 1.770; *p* = 0.19)^54^. An additional independent samples t-test showed no significant difference between mean learning gains in the experimental and control groups (Experimental group *M* = 0.087, *SD* = 0.074; Control group *M* = 0.112, *SD* = 0.096; *t*(71) = 1.226, *p* = 0.22). However, an analysis of variance (ANOVA) revealed that students in the experimental group learned significantly faster, irrespective of their prior knowledge of the content.

In Tang et al.^41^, 80 volunteer 10th-grade students were arbitrarily divided into two groups and had to complete a pre-test, a training session, and a post-test over the winter holiday. Sixty-five of them completed the study. The experimental group (*n* = 28) used *Guided and Adaptive Tutoring Tips* (GATT) within a *Mathematics Intelligent Assessment and Tutoring System* (MIATS) created by the authors, which provides step-by-step prompts and immediate personalized feedback on each incorrect question from the pre-test during the training sessions. The control group (*n* = 37) used another platform that provided only regular answer-based feedback, indicating whether their answers were correct or not. In the pre-test, the control group obtained a significantly higher average score than the experimental group, which aligned with the final examination scores from the previous semester. The mean difference between the treatment and control groups was −11.06, demonstrating statistical significance in an independent sample t-test (*p* = 0.0023 < 0.01). In the post-test, there was no statistically significant difference between the two groups (*p* = 0.113 > 0.01). The effect size indicated a small to moderate difference (Cohen’s *d* = 0.340). Between the tests, both groups made statistically significant progress (*p* < 0.01) in a paired sample t-test. The treatment group’s score increased by 15.50%, while the control group’s score increased by 5.13%. Compared to the pre-test, the treatment group showed greater progress in the post-test than the control group, which, according to the authors, indicates that the use of the intelligent teaching system significantly benefited the students in the treatment group.

Uriarte-Portillo^48^ conducted a study with 106 middle school students to compare an intelligent tutoring system with augmented reality (ARGeoITS) and a system with only augmented reality (ARGeo). Students were randomly assigned to the control group (*n* = 53) using ARGeo or the experimental group (*n* = 53) using ARGeoITS. In the first session of the experiment, all students received a lesson on basic geometry, a tutorial on augmented reality, and a pre-test. In the second session, students used a tablet with their respective platform for 50 minutes and then answered a post-test. The ANOVA test revealed that there was no statistically significant difference between the groups on the pre-test (F(1,106) = 0.182, *p* = 0.670). For the post-test, the mean achievement score was higher in the experimental group (*M* = 7.47, *SD* = 1.601) compared to the control group (*M* = 6.83, *SD* = 1.424), and the ANOVA showed a statistically significant difference (F(1,106) = 4.752, *p* = 0.032). This result indicates a better learning outcome for students using the ITS version of the learning platform. The authors also compared the post-test results according to the type of school the students attended (public or private). The ANOVA revealed a statistically significant difference (F(1,106) = 6.675, *p* = 0.011), favoring students from private schools (*M* = 7.62, *SD* = 1.396) compared to those from public schools (*M* = 6.84, *SD* = 1.566).

#### ITS vs modified ITS

This experimental design category includes eleven studies that compared one ITS to another ITS or a modified version of the same ITS. Identifying general trends in this category is challenging as the studies either compare ITSs with each other or with pedagogical methods for using an ITS in specific contexts. Only three studies in this category provide effect sizes, mostly small-sized^35,49,50^.

In Huang et al., 60 students used a redesigned ITS, while 69 students used the original version in high school Algebra 1 classes for a month, accumulating a total use of 320 min^50^. The redesigned ITS estimates the number of opportunities each student is likely to need to master easy and hard fine-grained knowledge contents, as to avoid under- or over-practice of a content. This feature was found to produce a significant improvement in learning, albeit with a small effect size. According to the authors, after an independent sample t-test, the redesigned version resulted in significantly higher learning gains with a small effect size (*b* = 0.05, *p* = 0.046; Cohen’s *d* = 0.31)^50^. Authors highlighted the relevance of data-driven redesign of ITSs to enhance their effectiveness.

In one of the studies conducted by Cui et al. [b], 46 students used Yixue in the experimental condition, while 58 students used BOXFiSH in the control condition^28^. The study aimed to investigate the effectiveness of these two language learning apps in teaching basic English grammar to foreign language learners over a two-day period. The results showed that the Yixue users performed significantly better than the control group, with 4.62 greater learning gains (Experimental group *M* = 5.86, *SD* = 10.81; Control group *M* = 1.04, *SD* = 8.93)^28^. The authors mention that these results might be due to Yixue’s fine granularity of knowledge contents. However, both groups showed improvement from pre-test to post-test. When interpreting the results of this particular study, it is important to remind that the main author is affiliated with the ITS company.

Long and Aleven conducted a randomized experiment to investigate the effectiveness of Cognitive Tutor in teaching geometry^44^. The study involved 47 students in the control group who used Cognitive Tutor with a control diary consisting of general questions, and 48 in the experimental group who used Cognitive Tutor with a skill diary for self-assessment. The experiment was conducted over three class periods. The results of the study indicated that there was a significant difference in learning gains between the groups on the reproduction problems section of the post-tests, which were isomorphic to the problems in Cognitive Tutor. This was determined through a one-way ANOVA (*F*(1, 93) = 3.861, *p* = 0.052, η² = 0.040). However, there was no significant difference between the two groups on the transfer problems section (*F*(1, 93) = 0.056, *p* = 0.814, η² = 0.001)^44^. Results suggest that self-assessment prompts could also support self-regulated learning for students using the ITS.

Long and Aleven [a] conducted a 2 × 2 experiment with 98 eighth-grade students over three class periods^46^. The study tested how the following independent factors influenced students’ performance: whether students were shown their skill-level and their progress in problem types, and whether students were allowed to select their next problem from an incomplete level^49^. The experiment used an ITS built with Cognitive Tutor Authoring Tools. No statistically significant differences were found among the four conditions, and there was no significant improvement between the pre- and post-tests^46^. The authors suspected a ceiling effect and decided to run another experiment, in which Long and Aleven [b] used the same 2 × 2 model with 56 seventh-grade students over five class periods on linear equations^46^. This second study modified the condition of displaying progress information to students, with the aim of encouraging reflection and limiting self-assessment biases. This change aimed to improve the accuracy of skill-level and problem-type-level progress information. Although the sample size was small, with only 14 students per group, the authors observed a positive effect on learning gains due to the modified progress information^46^. Both groups that received progress information outperformed their peers, showing a medium to large effect size (η² = 0.078) according to a one-way ANOVA^46^. These results suggest that the ITS leads to greater learning gains when it encourages students to reflect on their own progress and abilities.

McCarthy et al. conducted a study in which 118 high school students in science class used the iSTART ITS for three two-hour sessions in the same week^33^. The students were divided into four groups, following a 2 × 2 design to test how two metacognitive supports implemented within the ITS, performance threshold and self-assessment, influenced students’ performance in understanding complex text^33^. An additional 116 students who did not receive iSTART training only took the pre- and post-test. Contrary to the three previous studies’ results which suggested that self-assessment enhanced the effectiveness of the ITS^44,46^, none of the experimental conditions in this study were reported to influence performance^33^. Afterward, the 118 iSTART training students were compared to the 116 students without any iSTART training. The authors stated that the treatment improved the quality of self-explanations but did not affect test performance^33^.

In Walkington and Bernacki’s study, three experiment conditions were used to teach mathematics concepts using Cognitive Tutor: a surface personalization condition (*n* = 35), a deep personalization condition (*n* = 35) and a control condition with no personalization (*n* = 36)^35^. The students were also grouped based on their level of engagement with their interests, which were then used to personalize the exercises. The authors reported t-tests on the number of correct first attempts and correct answers per minute in a post-test, along with their respective effect sizes. The results indicated that students who received deep personalization had more correct first attempts, with a small-sized effect, than those who received surface personalization when their degree of engagement with the interest was high (*d* = 0.39)^35^. The study found that students who received personalization had a higher rate of correct answers per minute compared to the control group, with a large-sized effect, but this effect was observed only among those with a higher level of engagement with their interests (*d* = 0.92)^35^. Additionally, students who received surface personalization had more correct first attempts, with a small-sized effect, than those who received deep personalization when their level of engagement with their interests was low (*d* = −0.43)^35^. The same ITS, Cognitive Tutor, was also tested in a study by Bernacki and Walkington^34^, where 150 eleventh-grade students in Algebra I used Cognitive Tutor for four months. Ninety-nine participants used a personalized version based on an interest survey, while 51 participants used the standard version^34^. Results suggest that this personalization significantly improved students’ performance on a teacher-administered algebra exam (β = 0.062, *p* = 0.045)^34^. These two studies’ results suggest that the personalization of the ITS is sufficient to improve students’ performance^34,35^.

Holstein et al. conducted a three-condition experiment with 286 middle school students who used the Lynnette ITS for a total of 60 min over two days^49^. The first experimental group used Lynnette along with the complete version of the Lumilo glasses, enabling the teacher to monitor their activities and progress in real-time. The second experimental group used Lynnette with a limited version of the Lumilo glasses, which shared less data with the teacher. The control group used only Lynnette. The full version of Lumilo had a small positive effect size on student performance compared to the control condition (*r* = 0.21) and compared to the limited version (*r* = 0.11). These results corroborated with the authors’ hypotheses that combining real-time teaching with AI, supported by the analytics of an ITS, would enhance the student’s performance and learning surpassing the effects of monitoring support alone and the effects of conventional methods in ITS classrooms^49^. Only these authors acknowledged the potential impact of novelty on the effectiveness of the ITS in facilitating learning.

Vest et al.^51^ conducted a study comparing two approaches to problem-solving in basic algebra among 167 middle school students. One group used only an ITS for practice, while the other worked with examples that required selecting self-explanations before problem-solving activities. Participants were recruited via an online database and word of mouth, comprising 57 sixth graders, 73 seventh graders, and 36 eighth graders (one unreported). The study examined different types of worked examples, with or without visual representations and warm-up activities. However, the authors found little impact on student performance. These four conditions were consolidated into a single experimental group (*n* = 134) for comparison with a control group (*n* = 33). In both conditions, students received immediate feedback on their responses and could request scaffolded hints from the tutor at any time. Pre-tests and post-tests assessed procedural and conceptual knowledge, with results analyzed accordingly. The findings were similar across both item types, showing no significant effect of the condition: students with higher pre-test scores performed better on post-tests (procedural: F(1, 161) = 48.8, *p* < 0.001; conceptual: F(1, 161) = 90.62, *p* < 0.001). However, when the number of problems solved was included as a covariate, students in the experimental group outperformed those in the control group on both procedural and conceptual post-tests (procedural: β = 0.28, F(1, 161) = 5.32, *p* = 0.022; conceptual: β = 1.23, F(1, 161) = 7.18, *p* = 0.008). This suggests that generating self-explanations provided greater learning benefits than simply solving a comparable number of problems.

Horvers et al.^42^ studied the use of an ITS platform already used daily by four schools in 5th grade, involving a total of 114 students. Two schools continued using it as usual for learning fraction simplification (control condition), while the other two added goal-setting prompts via the Learning Path app (experimental condition). The experiment lasted one week, with 55-min lessons each day. On the first day, students completed the pre-test and received their first instruction on simplifying basic fractions. On the second day, they learned to simplify mixed fractions; on the third day, they worked on simplifying complex fractions; and on the fourth day, all three topics were reviewed in an integrated repetition lesson. On the fifth day, students completed the post-test. The ANCOVA revealed that students in the co-regulation condition solved more problems (F(1,111) = 4.26, *p* = 0.041, partial η² = 0.037) and had higher accuracy (F(1,112) = 45.68, *p* < 0.001, partial η² = 0.290) than those in the control condition. This suggests that engaging in co-regulation and goal-setting practices can support monitoring in an ITS. Analyses also showed that the control condition had higher learning gains than the experimental condition for complex fractions (F(1,107) = 10.67, *p* = 0.001, partial η² = 0.091). However, similar learning gains were found for basic fractions (F(1,107) = 0.90, *p* = 0.345, partial η² = 0.008) and mixed fractions (F(1,107) = 2.24, *p* = 0.137, partial η² = 0.021). Thus, students in both conditions learned equally well on easy and intermediate topics, but for the most difficult topic, the control condition outperformed the experimental condition.

#### ITS/No control

The four studies in this category did not include a control condition in their experimental design.

In Özyurt et al., 81 students used UZWEBMAT for 32 h over eight weeks in their mathematics class^45^. At the end of the eight weeks, 26 of them were interviewed. UZWEBMAT personalized the students’ learning paths according to their learning styles. Of the interviewed students, 21 expressed that their learning was facilitated. According to the feedback provided, some students found it helpful to be directed to the content of a different learning style when they failed to complete an exercise, as it provided a different perspective. Additionally, 18 students reported that they were able to complete the assignments independently without the need for teacher assistance^45^. This study’s results remind others previously described, according to which personalization of the ITS can enhance its effectiveness^34,35^.

In the study conducted by Chen et al., gender differences in cognitive load were compared using pre- and post-tests and a cognitive load questionnaire among a group of 24 fourth-grade students who learned with Zenbo^47^. The results of a sample t-test showed that boys had rated significantly lower than girls, with medium-sized effects, for mental effort (*t* = 2.859, *p* < 0.05, *d* = 0.825), mental load (*t* = 2.335, *p* < 0.05, *d* = 0.674), as well as cognitive load (*t* = 2.844, *p* < 0.05, *d* = 0.872)^47^. Boys also outperformed girls in the post-tests, but not significantly^47^. This study is the only one specifically investigating gender differences in ITS effects on learning and performance. The authors mention that the gender differences might be due to the fact that new technologies are more interesting and engaging to boys, but more distracting to girls^47^.

In Roscoe et al., 113 students used Writing Pal, which teaches English as a first language, for an average of 16 h over a six-month period^27^. The participants wrote an essay in November and another in May on two similar SAT prompts. Both pre-and post-study essays were graded, and their essay scores increased significantly (*t*(112) = 5.85, *p* = 0.001, *d* = 0.71)^27^. The authors also noted that positive changes were observed in essay structure and lexical sophistication^27^.

In Khasawneh 2024, 300 high school students from various grades and three schools used an ITS platform for eight weeks, integrated with their math instruction. A pre-test and post-test evaluation of three cognitive skills tools underwent thorough validation processes to confirm their reliability and validity by experts. After a pilot test with 50 students, internal consistency was assessed using Cronbach’s alpha coefficient (α = 0.80). Paired samples t-tests compared pre-test and post-test scores, revealing significant improvements in problem-solving (pre-test M = 65.4, post-test M = 72.8, t(299) = 4.67, *p* < 0.001), critical thinking (pre-test M = 68.9, post-test M = 74.3, t(299) = 3.82, *p* < 0.001), and logical reasoning abilities (pre-test M = 63.2, post-test M = 70.1, t(299) = 3.45, *p* = 0.001). An ANCOVA analysis also showed a significant positive impact of the intervention on post-test scores, controlling for pre-test scores, in problem-solving abilities (F(1, 298) = 10.21, *p* < 0.001), critical thinking abilities (F(1, 298) = 8.75, *p* < 0.001), and logical thinking abilities (F(1, 298) = 7.92, *p* < 0.001) after the intervention. These results suggest improvement after using adaptive learning technology in mathematics instruction and indicate a beneficial effect of the intervention on students’ cognitive ability enhancement.

## Discussion

This systematic review aimed to assess latest developments in ITS research and answer two research questions: 1) What experimental designs are used to evaluate the effects of ITSs? 2) What are the effects of ITSs on K-12 students’ learning and performance?

Several noteworthy observations emerged from this review. In contrast to Zawacki-Richter et al.‘s meta-analysis, in which a vast majority of the analyzed articles were written by authors with Computer Sciences or STEM backgrounds; the majority of analyzed articles in this review involved researchers in the field of educational sciences^12^. This is encouraging, as it enhances the reliability of the results regarding student learning and performance. The annual number of publications since 2009 has remained relatively constant, though modest. The United States and Asia, particularly Taiwan, China, Thailand, Korea and Turkey, are the primary locations for research on ITSs. None of the surveyed articles addressed ethical issues, which is a concerning oversight given recent advancements in artificial intelligence and associated ethical concerns.

Our analysis revealed that studies predominantly occurred in middle and high schools, with a particular emphasis on STEM fields, followed by languages. Holmes and Tuomi^12^ and UNESCO^11^ have highlighted that certain educational aspects within disciplines such as STEM facilitate research in these fields. The designs used are primarily quasi-experimental, involving experimental and control groups with pre- and post-tests to measure effects. The wide range of intervention durations, with many being very short, may amplify the effect size due to the novelty effect. Only Holstein et al. addressed this potential bias, which appears to be oversighted in this research field^49^.

This review presented the various effects on learning and performance documented in 26 publications. In comparison between an ITS and teacher-led instruction, seven out of eight articles reported a positive effect in favor of the ITS, with a medium to large size effect. Choi observed that the effect of ITS varied based on the educational level of participants^31^. It is difficult to draw a general conclusion from these studies’ results, since the ‘traditional teaching methods’ cannot necessarily be compared among all studies, and since the subject matter also varies.

When comparing an intelligent to a non-intelligent system, the results were more contradictory. Only one of four studies showed an advantage for ITS^37^, while the other three found no significant difference^32,36^. When comparing different types of ITSs or different versions of an ITS, the research tends to identify optimal conditions for using one ITS over another or for using ITSs with different pedagogical modalities.

Given the challenge of identifying general trends in this category and even more so to identify specific effects on learning and performance, we synthesized the information embedded in the current review sample to disentangle optimal conditions from the core components and features required for a successful ITS deployment that incorporates both technology tool use and encourages higher-order thinking skills.

Regarding the core components of an ITS, personalization and adaptivity appear to be part of a core of ITS components, which influence their effectiveness. An ITS that tailors instructional content based on individual student needs, prior knowledge, and learning styles tends to produce better learning outcomes. Studies have demonstrated that personalized learning paths, such as those employed by Yixue^28^ and UZWEBMAT^45^, can enhance learning gains by dynamically adjusting content difficulty and scaffolding instruction based on real-time performance. Another component is the capacity to provide immediate, real-time, data-driven feedback essential for reinforcing learning during task time. Unlike traditional pedagogical methods, where feedback may be delayed, a ITS that provides real-time guidance can assist students in identifying and correcting errors instantly. Systems such as Rimac^32,33,53,54^ and TECH8^30^ have shown how adaptive feedback mechanisms can enhance comprehension and accelerate learning. Integrating these components into an ITS has the advantage of breaking down complex concepts into manageable steps individualized to each learner, supplying just-in-time hints to reinforce learning and potentially improving student engagement and retention.

Outside of these core components, the features that have the potential to increase the acceptance, efficacy and utilization of ITS are those that, in combination, resolve pedagogical concerns and provide mechanisms for oversight. Such as providing the facility to support blended learning models that integrate teacher involvement^38,49^ and those supporting mastery-based learning with self-regulated learning strategies. While ITS can provide individualized instruction, they are reportedly most effective when combined with teacher-led guidance. Studies, such as those on WeWrite^38^ and Lynnette with Lumilo support^49^, emphasize that ITS should be considered complementary tools rather than replacements for educators. Furthermore, mastery-based learning support provides a foundation upon which students can build progress toward more complex topics only once they have demonstrated proficiency in prerequisite concepts, thereby helping to reduce learning gaps and equalizing negative perceptions concerning learning progress between learners. Additionally, ITS that incorporate self-regulation features, such as Cognitive Tutor’s skill diary^44^, encourage students to assess their own progress and take greater ownership of their learning. Self-regulation methods foster metacognitive skills, allowing students to monitor and adjust their strategies for more effective learning. The results from our sample indicate ITS that encourage self-assessment, such as those incorporating skill-level tracking or reflection prompts, improve self-regulation skills, leading to improved learning outcomes.

Taken as a whole and for a given metric, the success of ITS integration into an education ecosystem is contingent upon several interrelated factors, including student engagement, sustained use, and individual learner characteristics. An ITS that integrates gamification, real-world applications, and interactive elements can increase motivation, providing a deep personalization that enhances student interest and investment in learning. The benefits of ITS upon learners also appear to increase with prolonged exposure, as repeated interactions allow students to internalize concepts more effectively, aligning with broader pedagogical findings on the benefits of structured, long-term learning^31,33^. With reference to educators, repeated exposure or, more correctly, familiarization with an ITS’ components and features over time can lead to improved learning outcomes, as indicated by Pane and colleagues with CTAI^21^. However, the effectiveness of an ITS can vary based on learner individual factors such as prior knowledge, cognitive load, gender, and developmental stage, with lower-performing students often exhibiting greater benefits due to the tailored scaffolding these systems provide^47^. Importantly, middle school students frequently demonstrate more pronounced learning gains than their high school counterparts, highlighting the importance of deploying ITS with differentiated instructional designs^55,56^ based on sound pedagogical principles and methods.

Overall, our results suggest that ITSs may indeed enhance students’ learning and performance in certain conditions and considering certain modalities highlighted by research. Nevertheless, effects reported by analyzed studies are somewhat mitigated, and are hard to generalize due to the great variations in experimental designs.

In conclusion, given that ITSs are considered one of the most extensively researched AI applications for education according to UNESCO, the limited quantity of articles found for this review was somewhat unexpected^15^. However, the scope of this review may have been limited by the selection of databases, criteria for inclusion and exclusion, and publication and reporting bias. Further research is needed to evaluate the effects of ITSs on learning and performance in primary and secondary (K-12) education.

Future research should assess the effects of ITSs with longer interventions, larger and more diverse sample sizes including younger students, and better experimental control. For instance, it would be relevant for future research to consider the potential bias related to the novelty effect of using an ITS by utilizing longer interventions to assess this effect, such as in the longitudinal study by Pane and colleagues^21^. It may also be relevant to conduct further research comparing the effects of ITSs with traditional teaching methods, especially in non-STEM subjects. The oftentimes instructional nature of ITSs is frequently linked to a teaching approach centered around knowledge transmission, typically seen in STEM subjects^15^. This approach can be viewed as traditional and not in line with modern trends, such as collaborative teaching approaches^57^. However, AIEd as a domain and, more specifically, ITS as the front end of AIEd are progressing rapidly through constant innovation. The promise of AI to revolutionize education is predicated on its ability to provide adaptive and personalized learning experiences, thereby recognizing and nurturing the unique cognitive capabilities of each learner. In this regard, ITSs within the current sample appear to be well advanced toward providing this utility. More broadly, it would appear that integrating ITS with pedagogical approaches and practice presents unparalleled opportunities for personalized learning, efficiency, global reach, and the democratization of education that were previously unattainable through traditional educational approaches.

Regarding AI ethics in the deployment of ITS, the current sample of reviewed articles largely overlooked ethical considerations. This oversight highlights the need for scholars, researchers, and ITS implementers to draw from the rich AIEd literature related to the ethical concerns surrounding the multiple dimensions associated with deploying AI-based solutions within the education ecosystem. Finding the optimal balance between the benefits of AI in education and addressing the ethical challenges it poses is essential to deliver on the promise and potential of ITSs.

Given the exponential progress in generative AI, ITS integrating this technology will soon begin to emerge. Generative AI and now “thinking” models will become prevalent in education, providing fertile ground for new innovative research that investigates interactions with this new agentic software artifact, with an unprecedented ability to adapt educational content based upon natural interaction type interfaces.

Over a decade ago, VanLehn^13^ performed a review and comparative analysis of ITS and human tutoring. The review at the time challenged the assumption that human tutoring was vastly superior to computer-based tutoring. However, he illustrated that step-based and sub step-based ITS were able to achieve comparable effectiveness, with effect sizes ranging from 0.75 to 0.80. His findings brought to focus the interaction plateau hypothesis, which posits that while increasing interaction granularity enhances effectiveness, a plateau is reached, beyond which further refinement yields diminishing returns. Contrary to earlier estimates of a 2.0 sigma effect size, human tutoring exhibited a more modest effect size of 0.79. This reinforced the notion that ITS can serve as a viable, cost-effective alternative, particularly in large STEM courses (as demonstrated by Pane et al.). According to VanLehn, both human tutors and ITS can contribute to learning by providing feedback and scaffolding that can facilitate self-reflection and improve students’ understanding, a mechanism integral to problem-solving and conceptual mastery. Even with the mixed results from our current review sample, it would appear that the insights from VanLehn’s review are born out in the current ITS landscape. Therefore, given their demonstrated efficacy, ITS should be leveraged to supplement rather than replace classroom instruction, providing learning experiences in parallel to or outside direct teacher guidance^45^. UNESCO has recognized the global challenge of teacher shortages and high attrition rates, resulting in overcrowded classrooms and overburdened educators^58^.

The overarching conclusion to this review is that AI and teachers can collaborate effectively to optimize and facilitate student learning^38,49^. It is our position that AI can and should be used to support the learning experience of future generations and equip educators with tools to enrich their teaching capabilities.

## Methods

This systematic review adhered to the *Preferred Reporting Items for Systematic Reviews and Meta-Analyses* (PRISMA) guidelines^59,60^.

First, keywords related to our research topic were identified (scoping). Second, all retrieved articles were screened using the selected keywords to determine which ones to include in the systematic review (screening). Finally, the relevant data from the selected articles was extracted (extraction). The following subsections provide a detailed description of the scoping, screening and extraction methods followed by the PRISMA flow diagram.

### Scoping method

A web search was conducted using the keywords ‘Education’ and ‘AI’ to identify targeted keywords associated with ITSs, education, and learning at the primary and secondary school levels, as well as keywords associated with learners. ERIC USDE (Education Resources Information Center U.S. Department of education) and Scopus, two commonly used databases in education, were targeted. ERIC USDE specifically targets education-related papers, while Scopus is a more general database that lists computer science papers. The review focused on general education and user-related synonyms. Subsequently, specific synonyms of ITSs and AI within the education field were identified.

Domain-Specific Keywords (Education): Table 2 shows the first few papers sorted by relevance from the initial search in each of the two databases. We screened these papers to identify new synonyms and found eight for the domain. In the second search, we only identified one new synonym using those from the previous step, so we proceeded with the search for ITSs and AI specific keywords.

**Table 2:** Domain-specific (Education) keywords scoping results

	First search			Second search		
Database	Number of papers retrieved	Number of papers screened	New potential synonyms scoped	Number of papers retrieved	Number of papers screened	New synonyms scoped
ERIC	4761	7	Learning; Class; Pedagogy; Teaching; Commitment; Proficiency; Beneficial; Improvement; Motivation	37,804	4	Performance
Scopus	2,207,394	2		15,156	3	

Domain-Specific Keywords (ITSs and AIEd): Table 3 shows the initial search which was broad to gather as many relevant synonyms as possible. The second search was conducted using the six newly scoped synonyms. No additional synonyms were identified from the first few papers screened in the second search.

**Table 3:** Domain-specific (ITSs and AIEd) keywords scoping results

	First search			Second search		
Database	Number of papers retrieved	Number of papers screened	New potential synonyms scoped	Number of papers retrieved	Number of papers screened	New synonyms scoped
ERIC	775	6	ITSs; learning systems; technology enhanced; adaptive learning technology; e-learning	38	5	–
Scopus	1437	2		725	6	

Final query: The final query included the terms listed in Table 4. Some synonyms were excluded due to their tendency to produce irrelevant results. For instance, the term ‘ITS’ was removed because it generated numerous articles related to health issues (ITS is the French acronym for ‘infection transmise sexuellement’ or sexually transmitted disease). A filter was used to limit the results to publications released after 2009.

**Table 4:** Final query

Domain	Keywords
[Education] AND	[Education OR Classroom OR Teaching OR Pedagogy]
[ITS] AND	[“Tutoring system” OR “Tutoring systems” OR “Intelligent tutoring” OR “Adaptive learning technology” OR “Computer-based tutoring” OR “Computer tutor” OR “Automated tutoring”]
[Performance] AND	[Proficien* OR Beneficial OR Improvement OR Effectiv* OR Performance]
[K12]	[K12 OR Secondary OR Primary OR “High school” OR “Elementary school” OR “Middle school” OR “Primary school”]

The asterisk (*) is a boolean search operator for truncation.

Papers identified with this final query were downloaded into Covidence^61^, and the duplicates were automatically removed.

### Screening method

The initial stage of the screening process involved establishing Inter-Rater Reliability (IRR) between two screeners^62^. This was achieved by having both screeners review the title and the abstract of at least 50 randomly selected articles from the research bank and ensuring that there was less than 25% conflict (IRR > 75%). If the IRR was over 75%, both screeners proceeded to screen the articles, and both had the final say on whether to include the articles until the screening phase was completed. If there were any conflicting articles during IRR testing, they were reintegrated into the next step. If the IRR was below 75%, screeners received additional training and restarted the IRR process.

To expand our scope, we added a snowballing step using the most relevant articles sorted by Covidence^61^. The top 20 articles were imported into ResearchRabbit^63^, and all articles linked to two or more original articles (*n* = 39) were included in the review.

Inclusion Criteria: The review only included studies that focused on ITSs. To determine whether an article was about an ITS, the definition used during the screening process was taken from a meta-analysis on ITS^27^.

*Intelligent tutoring systems (ITS) are computer-assisted learning environments created using computational models developed in the learning sciences, cognitive sciences, mathematics, computational linguistics, artificial intelligence, and other relevant fields. ITS often are self-paced, learner-led, highly adaptive, and interactive learning environments operated through computers. ITS are adaptive in that they adjust and respond to learners with tasks or steps to suit learners’ individual characteristics, needs, or pace of learning. (Steenbergeen-Hu & Cooper, 2014, p. 970)*

For this review, only peer-reviewed and empirical research published in English between 2009 and January 14^th^ 2025 (when the search was conducted) was considered. The year 2009 was selected to provide an overview of the literature from the previous decade prior to the emergence of Covid-19. The studies had to focus on students in grades K–12 within a formal school context. Formal school contexts refer to educational institutions that deliver certifications or degrees as part of their official educational systems, as opposed to informal or non-formal education.

Exclusion Criteria: Research focusing exclusively on students with learning disabilities, social impairments, or emotional disorders (e.g., students with attention-deficit/hyperactivity disorder) were excluded to ensure generalization of findings to a broader population.

### Extraction method

The extraction process involved identifying relevant information in each selected article and recording in an extraction codebook within Covidence^61^. This codebook listed the specific elements to address our research questions: title; authors’ affiliation; mention of AI ethics; study design; country; population description; school level; school subject; total number of participants; study aim; controlled variable; dependant variables; duration; results; analysis, limitations, and conclusions; and future research avenues.

To establish Inter-Rater Reliability (IRR) between two screeners, the first extraction step required two extractors to independently complete the extraction process for 10 randomly selected articles from the retrieved bank^62^. The goal was to maintain less than 25% conflict (IRR > 75%). Once the extraction process was completed, a lead screener compared the answers of the two screeners and asserted the IRR. If the IRR was too low, the lead screener provided feedback and training to the two original screeners. In the current case, the IRR stayed above 75%, this process was not needed.

### Data analysis

The extracted data was initially organized to provide an overview of the articles based on: authors’ affiliations; country; school level; school subject; intervention duration; participants; date of publication; and mention of AI ethics. Zawacki-Richter et al.’s systematic review regarding AI applications in higher education inspired the elements of the extraction codebook^12^. The data was subsequently analyzed based on the research questions. For this phase of the analysis, we categorized studies based on their experimental design.

### Initial limitations

This review focused exclusively on articles written in English within the fields of education and computer science. Restricting the selection to only two databases might also have limited the scope of this review. In addition, it is imperative to consider publication and reporting bias^64^. Therefore, any systematic review may be more likely to report positive and significant effects of ITSs. Finally, this systematic review focused specifically on the effects of ITSs on learning and performance. Other educational variables, such as interest, attitude or motivation towards school subjects, were not considered in this review but should be considered in future research.

### PRISMA flow diagram

**Fig. 2:**
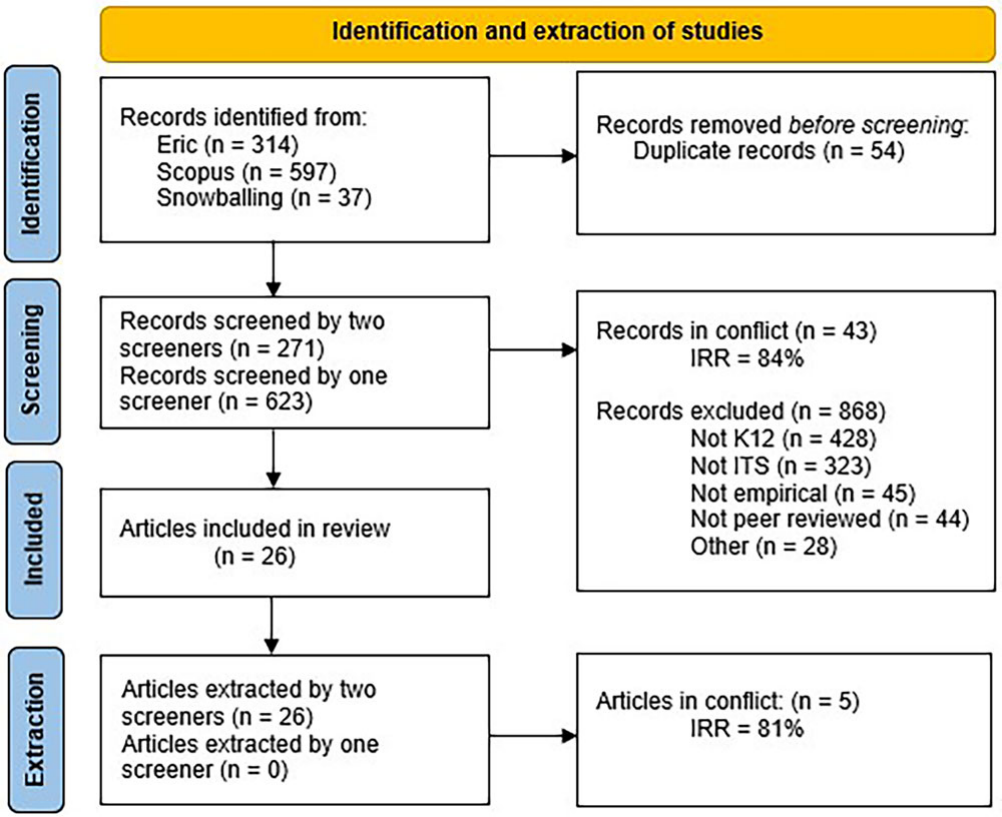
PRISMA flow diagram.

The PRISMA Flow Diagram, shown in Fig. 2, presents the results of the identification and extraction of the studies. A total of 948 records were retrieved in Eric and Scopus, as well as through the snowballing process. After removing 54 duplicates, 868 records were excluded during the screening process as they failed to meet the inclusion criteria. Finally, 26 records were included in the review. It is important to note that two of the included records, namely Cui et al.^28^ and Long and Aleven^46^, presented two different studies about ITSs in one article^28,46^. Therefore, although the total number of articles was 26, the total number of studies was actually 28.

## Data Availability

Data sharing is not applicable to this article as no datasets were generated or analyzed during the current study.

## References

[1] UNESCO. *Artificial Intelligence in Education: Challenges and Opportunities for Sustainable Development* (UNESCO, 2019).

[2] UNESCO. *Beijing Consensus on Artificial Intelligence and Education - UNESCO Bibliothèque Numérique*. in 70 (UNESCO, 2019).

[3] UNESCO. *Global Education Monitoring Report 2023: Technology in Education: A Tool on Whose Terms?*10.54676/UZQV8501 (GEM Report UNESCO, 2023).

[4] Ferrari, A., Punie, Y. & Redecker, C. Understanding Digital Competence in the 21st Century: An Analysis of Current Frameworks. in *21st Century Learning for 21st Century Skills* (eds. Ravenscroft, A., Lindstaedt, S., Kloos, C. D. & Hernández-Leo, D.) 79–92 10.1007/978-3-642-33263-0_7 (Springer, Berlin, Heidelberg, 2012).

[5] OECD. *Education at a Glance 2015: OECD Indicators* (Organisation for Economic Co-operation and Development, 2015).

[6] Godwin-Jones, R. Emerging Technologies - Challenging Hegemonies in Online Learning. *Lang. Learn. Technol.* **16**, 4–13 (2012).

[7] Kulik, J. A. *Effects of Using Instructional Technology in Elementary and Secondary Schools: What Controlled Evaluation Studies Say* (Sri International, 2003).

[8] Picciano, A. G. The Evolution of Big Data and Learning Analytics in American Higher Education. *J. Asynchronous Learn. Netw.* **16**, 9–20 (2012).

[9] Winkler, R. & Soellner, M. Unleashing the Potential of Chatbots in Education: A State-Of-The-Art Analysis. *Acad. Manag. Proc.* **2018**, 15903 (2018).

[10] Vaswani, A. et al. Attention is All you Need. In: *31st Conference on Neural Information Processing Systems* (2017).

[11] Mhlanga, D. Open AI in Education, the Responsible and Ethical Use of ChatGPT Towards Lifelong Learning. SSRN Scholarly Paper at 10.2139/ssrn.4354422 (2023).

[12] Zawacki-Richter, O., Marín, V. I., Bond, M. & Gouverneur, F. Systematic review of research on artificial intelligence applications in higher education – where are the educators? *Int. J. Educ. Technol. High. Educ.* **16**, 39 (2019).

[13] VanLehn, K. The Relative Effectiveness of Human Tutoring, Intelligent Tutoring Systems, and Other Tutoring Systems. *Educ. Psychol.* **46**, 197–221 (2011).

[14] Duolingo Inc. (Duolingo (Version 5.140.4) [Android, iOS]. https://www.duolingo.com/ (2024).

[15] UNESCO. *Artificial Intelligence and Education. Guidance for Policy-Makers*. *United Nations Educational, Scientific and Cultural Organization**(UNESCO): Paris, France*. https://unesdoc.unesco.org/ark:/48223/pf0000376709 (2021).

[16] Holmes, W. & Tuomi, I. State of the art and practice in AI in education. *Eur. J. Educ*. **57**, 542–570 (2022).

[17] Kulik, J. A. & Fletcher, J. D. Effectiveness of Intelligent Tutoring Systems: A Meta-Analytic Review. *Rev. Educ. Res.* **86**, 42–78 (2016).

[18] Holmes, W., Anastopoulou, S. & Mavrikis, E. Technology-enhanced. In: *Personalised Learning: Untangling the Evidence* (Stuttgard: Robert Bosch Stiftung, 2018).

[19] Mello-Carpes, P. B. *IBE — Science of Learning Portal — Novelty as a Strategy to Improve Learning,* https://solportal.ibe-unesco.org/articles/novelty-as-a-strategy-to-improve-learning/ (2020).

[20] Honebein, P. C. & Reigeluth, C. M. To prove or improve, that is the question: the resurgence of comparative, confounded research between 2010 and 2019. *Educ. Technol. Res. Dev.* **69**, 465–496 (2021).

[21] Pane, J. F., Griffin, B. A., McCaffrey, D. F. & Karam, R. Effectiveness of Cognitive Tutor Algebra I at Scale. *Educ. Eval. Policy Anal.* **36**, 127–144 (2014).

[22] Smith, S. G. & Sherwood, B. A. Educational Uses of the PLATO Computer System. *Science* **192**, 344–352 (1976). 769165 10.1126/science.769165

[23] Adams, C., Pente, P., Lemermeyer, G. & Rockwell, G. Ethical principles for artificial intelligence in K-12 education. *Comput. Educ. Artif. Intell.* **4**, 100131 (2023).

[24] Ali, S. et al. Explainable Artificial Intelligence (XAI): What we know and what is left to attain Trustworthy Artificial Intelligence. *Inf. Fusion* **99**, 101805 (2023).

[25] Holmes, W. et al. Ethics of AI in Education: Towards a Community-Wide Framework. *Int. J. Artif. Intell. Educ.* **32**, 504–526 (2022).

[26] Roscoe, R. D. & McNamara, D. S. Writing Pal: Feasibility of an Intelligent Writing Strategy Tutor in the High School Classroom. *J. Educ. Psychol.* **105**, 1010–1025 (2013).

[27] Roscoe, R. D., Allen, L. K., Weston, J. L., Crossley, S. A. & McNamara, D. S. The writing pal intelligent tutoring system: Usability testing and development. *Comput. Compos.* **34**, 39–59 (2014).

[28] Cui, W., Xue, Z. & Thai, K.-P. Performance Comparison of an AI-Based Adaptive Learning System in China. In: *Proceedings of the Chinese Automation Congress (CAC)*, 3170–3175 (Institute of Electrical and Electronics Engineers Inc., 2019). 10.1109/CAC.2018.8623327.

[29] Chen, H.-R. & Huang, H.-L. Learning achievement of knowledge management adaptivity in web-based interactive learning systems for a junior high school in Taiwan. *N. Educ. Rev.* **25**, 183–193 (2011).

[30] Dolenc, K., Aberšek, B. & Aberšek, M. K. Online functional literacy, intelligent tutoring systems and science education. *J. Balt. Sci. Educ.* **14**, 162–171 (2015).

[31] Choi, I.-C. Efficacy of an ICALL Tutoring System and Process-Oriented Corrective Feedback. *Comput. Assist. Lang. Learn.* **29**, 334–364 (2016).

[32] Jordan, P., Albacete, P. & Katz, S. *Adapting Step Granularity in Tutorial Dialogue Based on Pretest Scores*, 148 (Springer Verlag, 2017).

[33] McCarthy, K. S., Jacovina, M. E., Snow, E. L., Guerrero, T. A. & McNamara, D. S. *iSTART Therefore I Understand: But Metacognitive Supports Did Not Enhance Comprehension Gains* (Grantee Submiss, 2017).

[34] Bernacki, M. L. & Walkington, C. The Role of Situational Interest in Personalized Learning. *J. Educ. Psychol.* **110**, 864–881 (2018).

[35] Walkington, C. & Bernacki, M. L. Personalizing Algebra to Students’ Individual Interests in an Intelligent Tutoring System: Moderators of Impact. *Int. J. Artif. Intell. Educ.* **29**, 58–88 (2019).

[36] Katz, S. et al. Linking Dialogue with Student Modelling to Create an Adaptive Tutoring System for Conceptual Physics. *Int. J. Artif. Intell. Educ.* **31**, 397–445 (2021).

[37] Ingkavara, T., Panjaburee, P., Srisawasdi, N. & Sajjapanroj, S. The use of a personalized learning approach to implementing self-regulated online learning. *Comput. Educ. Artif. Intell.* **3**, 100086 (2022).

[38] Wijekumar, K. K., Harris, K. R., Graham, S. & Lei, P. A Teacher Technology Tango Shows Strong Results on 5th Graders Persuasive Writing. *Educ. Technol. Res. Dev.* **70**, 1415–1439 (2022).

[39] Borchers, C. et al. In: Responsive and Sustainable Educational Futures. EC-TEL 2023. (eds Viberg, O., Jivet, I., Muñoz-Merino, P., Perifanou, M., Papathoma, T) Vol. 14200 *What Makes Problem-Solving Practice Effective? Comparing Paper and AI Tutoring,* 44–59 Vol. 14200 (Springer, Cham, 2023).

[40] Nehring, J., Moyer-Packenham, P. & North, M. Assessing the effectiveness of an artificial intelligence tutoring system for improving college-level mathematics preparedness in high school students. *Issues Inf. Syst.* **24**, 128–141 (2023).

[41] Tang, R., Zhang, Y., Cao, Y., Liu, H. & Jia, J. *Design and Effect of Guided and Adaptive Tutoring Tips for Helping School Mathematics Problems Solving*, 273–284 (2023).

[42] Horvers, A. et al. How does co-regulation with Adaptive Learning Technologies affect primary school students' goal-setting, regulation of practice behavior and learning outcomes?. *Front. Educ.* **9**, 1435483 (2024).

[43] Khasawneh, M. A. S. Implementing adaptive learning technologies: Practical strategies for enhancing cognition in mathematics education. *Int. J. Adv. Appl. Sci.* **11**, 111–118 (2024).

[44] Long, Y. & Aleven, V. *Skill Diaries: Can Periodic Self-Assessment Improve Students’ Learning with an Intelligent Tutoring System?* Lect. Notes Comput. Sci. vol. 7315 LNCS 674 (Springer 2012).

[45] Özyurt, Ö., Özyurt, H., Baki, A., Güven, B. & Karal, H. Evaluation of an adaptive and intelligent educational hypermedia for enhanced individual learning of mathematics: A qualitative study. *Expert Syst. Appl.* **39**, 12092–12104 (2012).

[46] Long, Y. & Aleven, V. *Active Learners: Redesigning an Intelligent Tutoring System to Support Self-Regulated Learning*, 495 (2013).

[47] Chen, B., Hwang, G.-H. & Wang, S.-H. Gender Differences in Cognitive Load When Applying Game-Based Learning with Intelligent Robots. *Educ. Technol. Soc.* **24**, 102–115 (2021).

[48] Uriarte-Portillo, A., Zatarain-Cabada, R., Barrón-Estrada, M. L., Ibáñez, M. B. & González-Barrón, L.-M. Intelligent Augmented Reality for Learning Geometry. *Inf. Switz*. **14**, (2023).

[49] Holstein, K., McLaren, B. M. & Aleven, V. In: J. Culbertson, A. Perfors, H. Rabagliati & V. Ramenzoni (Eds.), Proceedings of the 44th AnnualConference of the Cognitive Science Society. *Student Learning Benefits of a Mixed-Reality Teacher Awareness Tool in AI-Enhanced Classrooms,* 168 (Springer Verlag, 2018).

[50] Huang, Y. et al. A general multi-method approach to data-driven redesign of tutoring systems. In: *ACM International Conference on Proceedings Series,* 161–172 (Association for Computing Machinery, 2021). 10.1145/3448139.3448155.

[51] Vest, N. A. et al. *Self-Explanation of Worked Examples Integrated in an Intelligent Tutoring System Enhances Problem Solving and Efficiency in Algebra*. in 3466–3472 (2022).

[52] Chen, C.-S., Cheng, M.-Y. & Wu, Y.-W. Seismic assessment of school buildings in Taiwan using the evolutionary support vector machine inference system. *Expert Syst. Appl.* **39**, 4102–4110 (2012).

[53] Ökördi, R. & Molnár, G. Computer-Based Intervention Closes Learning Gap in Maths Accumulated in Remote Learning. *J. Intell*. **10**, 58 (2022). 10.3390/jintelligence10030058 PMC9397034 35997414

[54] Albacete, P. et al. The impact of student model updates on contingent scaffolding in a natural-language tutoring System. In: (eds S. Isotani, E. Millan, A. Ogan, P.Hastings, B. McLaren & Luckin, R) Artificial Intelligence in Education, 20th International Conference, AIED 2019, Chicago, IL, USA, Proceedings, Part II, 37–49. 10.1007/978-3-030-23204-7_4 (2019).

[55] Deunk, M. I., Smale-Jacobse, A. E., de Boer, H., Doolaard, S. & Bosker, R. J. Effective differentiation Practices:A systematic review and meta-analysis of studies on the cognitive effects of differentiation practices in primary education. *Educ. Res. Rev.* **24**, 31–54 (2018).

[56] Smale-Jacobse, A. E., Meijer, A., Helms-Lorenz, M. & Maulana, R. Differentiated Instruction in Secondary Education: A Systematic Review of Research Evidence. *Front. Psychol*. **10**, 2366 (2019). 10.3389/fpsyg.2019.02366 PMC6883934 31824362

[57] Jelovica, L. & Alajbeg, A. An Overview of the Characteristics of a Modern School. *Croat. J. Educ. Hrvat. Časopis Za Odgoj Obraz.* **25**, 1001–1031 (2023).

[58] UNESCO. Highlights from the global report on teachers: What you need to know. UNESCO, https://www.unesco.org/en/articles/highlights-global-report-teachers-what-you-need-know (2023).

[59] Page, M. J. et al. The PRISMA 2020 statement: an updated guideline for reporting systematic reviews. *BMJ* **372**, n71 (2021). 33782057 10.1136/bmj.n71 PMC8005924

[60] Page, M. J. et al. PRISMA 2020 explanation and elaboration: updated guidance and exemplars for reporting systematic reviews. *BMJ* **372**, n160 (2021). 33781993 10.1136/bmj.n160 PMC8005925

[61] Veritas Health Innovation. *Covidence systematic review software* (Veritas Health Innovation, 2023).

[62] Gisev, N., Bell, J. S. & Chen, T. F. Interrater agreement and interrater reliability: Key concepts, approaches, and applications. *Res. Soc. Adm. Pharm.* **9**, 330–338 (2013). 10.1016/j.sapharm.2012.04.004 22695215

[63] Human Intelligence Technologies Inc. ResearchRabbit [Software]. https://www.researchrabbit.ai/ (2023).

[64] Dwan, K. et al. Systematic Review of the Empirical Evidence of Study Publication Bias and Outcome Reporting Bias. *PLOS ONE* **3**, e3081 (2008). 18769481 10.1371/journal.pone.0003081 PMC2518111

